# Dry Eye Syndrome Risks in Patients With Fibromyalgia: A National Retrospective Cohort Study: Erratum

**DOI:** 10.1097/01.md.0000484179.78656.3b

**Published:** 2016-05-13

In the articles “Dry Eye Syndrome Risks in Patients With Fibromyalgia: A National Retrospective Cohort Study”,^1^ Dr. Ming-Chia Lin's affiliation was not reported in full. Dr. Lin is affiliated with the E-Da Hospital. The articles have since been corrected online.
